# Study of association of leptin with leukocyte telomere length in a Chinese rural population

**DOI:** 10.1186/s12944-024-02097-x

**Published:** 2024-04-13

**Authors:** Juan Li, Chan Yang, Yadi Zhang, Qingqing Li, Xiaowei Liu, Yuhong Zhang, Yi Zhao

**Affiliations:** 1 Public Health School, Ningxia Medical University, Yinchuan, 750004 China; 2 School of Nursing, Ningxia Medical University, Yinchuan, Ningxia China; 3 NHC Key Laboratory of Metabolic Cardiovascular Diseases Research, Ningxia Medical University, Yinchuan, 750004 China; 4 Key Laboratory of Environmental Factors and Chronic Disease Control, Ningxia Medical University, Yinchuan, Ningxia China

**Keywords:** Insulin resistance, Leptin, Telomere length, Interaction

## Abstract

**Background:**

Previous studies have demonstrated the relationship between adipocyte factors, insulin resistance, and other indicators with telomere length. However, these studies did not consider the influence of changes in different indicators on telomere length over time. Therefore, the aim of this study is to elucidate the impact of changes in adipocyte factors, HOMA-IR, and other indicators on the dynamic variation of telomere length.

**Methods:**

The data were from a cohort study conducted in Ningxia, China. A total of 1624 subjects were analyzed. Adipokines and relative leukocyte telomere length (RLTL) were measured, and changes in Homeostatic Model Assessment for Insulin Resistance (HOMA-IR), Homeostatic Model Assessment for β-Cell Function (HOMA-β), and Quantitative Insulin Sensitivity Check Index (QUICKI) were calculated. Generalized linear models evaluated associations between changes in adipokines and RLTL changes. Furthermore, univariate analyses examined the effects of changes in adipokines and insulin resistance indicators on ΔRLTL.

**Results:**

The research findings indicate that females generally have shorter telomeres compared to males. In comparison to the low-level group of Δleptin (LEP), the high-level group of ΔLEP shows a negative correlation with ΔRLTL (B=-1.32, 95% CI (-2.38, -0.27)). Even after multivariable adjustments, this relationship persists (B=-1.31, 95% CI (-2.24, -0.23)). Further analysis reveals that after adjusting for ΔHOMA-IR, ΔHOMA-β, and ΔQUICKI, the high-level group of ΔLEP still exhibits a significant negative correlation with ΔRLTL (B=-1.37, 95% CI (-2.43, -0.31)). However, the interaction effects between ΔHOMA-IR, ΔHOMA-β, ΔQUICKI, and ΔLEP do not affect ΔRLTL.

**Conclusions:**

Elevated levels of leptin were significantly correlated with shortened telomere length. This suggests that increased leptin levels may impact overall individual health by affecting telomere length, underscoring the importance of measures to reduce leptin levels to mitigate the onset and progression of related diseases.

**Supplementary Information:**

The online version contains supplementary material available at 10.1186/s12944-024-02097-x.

## Background

The telomere, a complex structure located at the ends of eukaryotic chromosomes, comprises repetitive non-coding DNA sequences and specific binding proteins [1]. Telomeres undergo shortening during cellular division, and critically shortened telomeres can instigate cellular apoptosis or senescence, thereby influencing cellular lifespan [2]. Moreover, epidemiological studies have elucidated correlations between telomere length and age-related ailments such as diabetes [3], cardiovascular disease [4] and others.

Adipose tissue, an indispensable endocrine and paracrine organ within the human body, secretes a plethora of hormones, growth factors, and cytokines collectively referred to as adipokines [5]. Among these, leptin and adiponectin are pivotal hormones secreted by adipocytes, playing crucial roles in physiological processes including insulin resistance and lipid metabolism. Additionally, their involvement in telomere shortening processes has garnered significant attention [6]. Despite the capability of adipose tissue to secrete various cytokines, the relationship between adipocytokines and leukocyte telomere length (LTL) remains underexplored. Only a limited number of studies have explored this association, yielding inconsistent findings. While some studies have indicated a positive correlation between adiponectin and LTL [7]. others have suggested an inverse relationship between leptin and telomere length, even after adjusting for confounders [8].

Emerging evidence suggests a correlation between telomere length and the pathogenesis of diabetes, with shorter telomeres being associated with the condition [9]. Insulin resistance represents a pathological state evident in the initial stages of numerous chronic metabolic disorders. However, the association between Homeostatic Model Assessment for Insulin Resistance (HOMA-IR), a pivotal indicator of insulin resistance, and leukocyte telomere length (LTL) exhibits inconsistency across studies [10–12], warranting further investigation to elucidate their relationship.

In summary, most studies primarily rely on cross-sectional data, while results from longitudinal studies typically focus solely on the impact of baseline indicators on subsequent telomere length, without considering the dynamic effects of changes in various indicators on telomere length variation. Therefore, the objective of this study is to explore the influence of alterations in adipokines, HOMA-IR, and other indicators on variations in telomere length. By scrutinizing numerical changes at distinct time intervals, novel temporal correlations will be unveiled, thus furnishing critical theoretical and practical underpinnings for the prevention of associated diseases.

## Methods

### Study design and subject selection

**Fig. 1:**
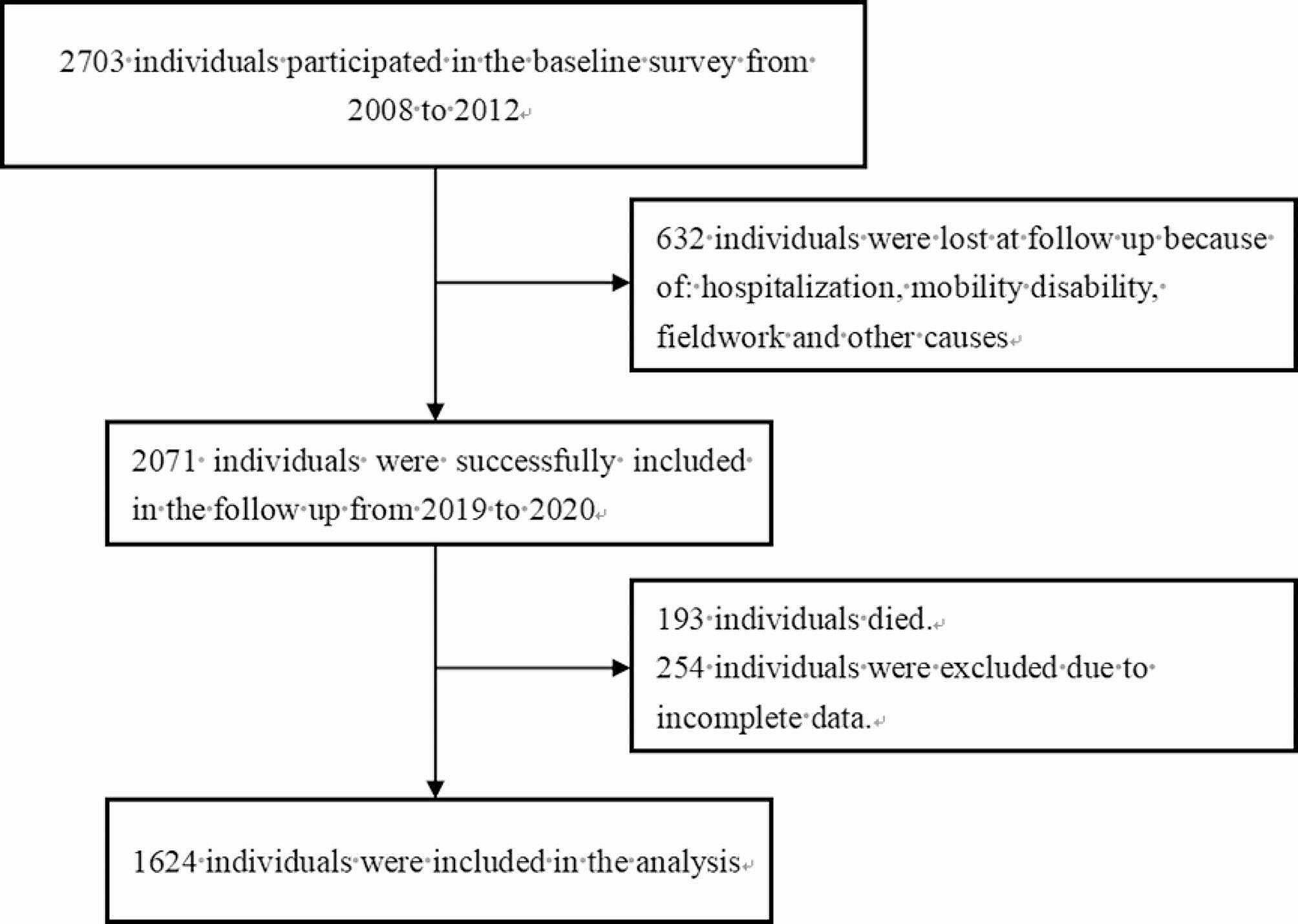
Flow chart of participant selection

The data were obtained from a cohort study conducted in northwestern Ningxia spanning from 2008 to 2012 to 2019–2020. Employing a stratified cluster sampling approach, a total of 2703 individuals aged 25–74 years were enlisted to partake in the questionnaire survey, anthropometric measurements, and biological sample collection [13]. A follow-up survey was executed between 2019 and 2020, involving 2071 participants, among whom 193 deaths were recorded. The average person-years of the study population amounted to 9.46 (minimum: 6.75, maximum: 12.17). Subsequently, 1624 individuals, comprising 654 males and 970 females, met the specified inclusion and exclusion criteria and were thus included for analysis (Fig. 1). Prior to their involvement, all participants provided signed informed consent forms. Ethical approval for the study was obtained from the Ethics Committee at Ningxia Medical University.

### Anthropometric measurements

Structured questionnaires were administered through face-to-face interviews subsequent to obtaining informed consent from the participants. These questionnaires encompassed demographic data such as age, gender, marital status, education level, lifestyle factors including smoking and alcohol consumption habits, as well as medical history. Height and weight were accurately measured to the nearest 0.1 cm and 1 kg, respectively, utilizing a height scale and weight scale. Blood pressure was assessed employing an OMRONHEM-801 electronic sphygmomanometer. Prior to measurement, participants rested for 5–10 min and maintained a seated position with their upper arm exposed at heart level. Two blood pressure readings were obtained with a 3–5 min interval between measurements, and the average was calculated.

### Biochemical measurements

Fasting blood samples were obtained following an overnight fast of 8 h. At baseline, fasting plasma glucose (FPG) levels were determined using One Touch Ultra 2 (Life Scan, USA), while serum insulin levels were assessed through enzyme-linked immunochromatographic assay. During the follow-up period, biochemical autoanalyzers (Mindray BS-430, Shenzhen, China) were utilized to measure total cholesterol (TC), triglycerides (TG), high-density lipoprotein cholesterol (HDL-C), and low-density lipoprotein cholesterol (LDL-C). Serum insulin (FINS) levels were quantified via chemiluminescence immunoassay using the Mindray CL-2000i system (Shenzhen, China). Additionally, leptin and adiponectin levels were determined using ELISA kits (CSB-E07270h, CSB E04649h, CUSABIO, China).

### DNA extraction and telomere length analysis

Genomic DNA was isolated from peripheral blood leukocytes using the DNA Blood Midi Kit (Bao Bioengineering, Japan) and quantified for concentration and purity via absorbance at 260/280 nm utilizing a Biospec-nano spectrophotometer (Shimadzu, Japan). Relative leukocyte telomere length (RLTL) was assessed using quantitative real-time PCR (Bio-Rad, Germany), following established protocols. Telomere (T) and 36B4 (S) gene sequences were amplified separately, each in their designated PCR plates, with the CT curves for each amplicon available in Figure S1 and Figure S2. Each plate included a reference gene reaction and a negative control. The PCR conditions for telomere amplification were as follows: initial denaturation at 95 °C for 10 min, followed by denaturation at 95 °C for 15 s, annealing at 54 °C for 2 min for 22 cycles. For 36B4, the conditions were similar except for the annealing temperature, which was set at 58 °C for 30 cycles. Subsequently, the relative T/S ratio, indicative of RLTL, was calculated utilizing the ΔΔCt method. The following equations were employed: T/S = [2^Ct(telomere)^ / 2^Ct(36B4^)]^−1^= 2^−ΔCt^, RLTL = 2^−ΔCt^(need checking sampling) / 2^−ΔCt^(reference gene) [12].

### Definition of variable

HOMA-IR was calculated using the following formulae:

HOMA-IR = [FINS(MU/L) × FPG (mmol/L)/22.5]; Quantitative insulin sensitivity check index (QUICKI) = 1 / (lg FINS µIU/mL + lg FPG mmol/L); Homeostatic model assessment of β cell (HOMA-β) = 20 × FINS µIU/Ml / (FPG mmol/L − 3.5) [14].

Changes in telomere shortening (ΔRLTL) = follow-up relative telomere length - baseline relative telomere length; ΔRLTL represents the change in relative leukocyte telomere length. A smaller ΔRLTL indicates a shorter relative change in leukocyte telomere length.

Changes in adiponectin (ΔADP) = follow-up adiponectin - baseline adiponectin.

Changes in leptin (ΔLEP) = follow-up leptin - baseline leptin.

Based on different levels of educational attainment, education is categorized into two types: low (junior high school and below) and high (high school and above).

Physical exercise: The definition of physical exercise is to exercise at least three times a week, with each session lasting at least 30 min.

### Statistical analysis

Analyses were performed using R version 4.2.2 and SPSS version 24.0 statistical software. For normally distributed data, continuous variables were described by mean ± standard deviation. For non-normally distributed data, using median with interquartile range. Categorical variables were described using frequency and percentage. T-tests and chi-square tests were employed to compare general characteristics between different genders. Additionally, Pearson correlation coefficient was used to analyze the correlation between LEP, ADP, and RLTL, with age-adjusted Pearson correlation analysis performed. In further analysis, generalized linear models (GLMs) with linear regression were applied, using the low level of change in adipocyte factors as a reference, to assess the relationship between adipocyte factors and RLTL. Specifically, four linear regression models were established: Model 1 - No adjustment; Model 2 - Model 1 + sex, age; Model 3 - Model 2 + education, smoking, drinking, exercise; Model 4 - Model 3 + BMI, SBP, DBP, FPG [12, 15, 16]. Finally, univariate analysis was employed to evaluate the interaction effects of HOMA-IR, HOMA-β, QUICI, and adipocyte factors on RLTL.

## Results

### Characteristics of the study population

In this study, we enrolled a total of 1624 subjects, with females comprising 59.7% of the sample. Participants were stratified into two groups based on gender. As indicated in Table 1, females exhibited significantly higher levels of BMI, TC, TG, HDL-C, LDL-C, ΔADP, and ΔLEP (25.5 ± 6.4, 5.0 ± 1.0, 1.8 ± 1.1, 1.8 ± 0.5, 3.9 ± 1.7, -8.7 ± 26.6, -0.4 ± 8.1, respectively) compared to males, except for TG and ΔHOMA-IR, where no statistically significant differences were observed between the sexes. Although females demonstrated higher levels of ΔRLTL compared to males, the disparity between the two groups did not attain statistical significance. Furthermore, among males, a higher proportion reported smoking (31.3%) and drinking (24.6%) habits. Conversely, females exhibited lower levels of blood glucose and diastolic blood pressure compared to males, yet these distinctions did not reach statistical significance.

**Table 1:** Comparison of the baseline characteristics among the different groups

Variables	All Subjects (*n* = 1624)	Female		Male		P	
		(*n* = 970)	(*n* = 654)				
Age (years)	57.6 ± 10.2	56.7 ± 10.0	58.9 ± 10.4		< 0.001		
Education (high) n (%)	476 (29.3%)	225 (23.2%)	251 (38.4%)		< 0.001		
Smoke yes) n %	213 (13.1%)	8 (0.8%)	205 (31.3%)		< 0.001		
Drink (yes) n %	212 (13.0%)	51 (5.3%)	161 (24.6%)		< 0.001		
Physical exercise (yes) n %	531 (32.7%)	340 (35.1%)	191 (29.2%)		0.015		
ΔHOMA-IR							
T1	1128 (69.5%)	635 (65.5%)	493 (75.4%)		< 0.001		
T2	496 (30.5%)	335 (35.5%)	161 (24.6%)				
ΔHOMA-β							
T1	846 (52.1%)	456 (47.0%)	390 (59.6%)		< 0.001		
T2	778 (47.9%)	514 (53.0%)	264 (40.4%)				
ΔQUICKI							
T1	1210 (74.5%)	751 (77.4%)	459 (70.2%)		< 0.001		
T2	414 (25.5%)	219 (22.6%)	195 (29.8%)				
BMI (kg/m^2^)	24.9 ± 6.4	25.5 ± 6.4	25.0 ± 3.4		0.055		
SBP (mmHg)	133.5 ± 27.4	136.7 ± 20.6	135.5 ± 19.1		0.238		
DBP (mmHg)	84.0 ± 18.1	85.6 ± 14.1	85.8 ± 13.2		0.829		
FPG (mmol/L)	5.6 ± 2.2	5.8 ± 1.7	5.9 ± 2.3		0.427		
TG (mmol/L)	1.6 ± 1.1	1.8 ± 1.1	1.6 ± 1.1		0.057		
TC (mmol/L)	4.7 ± 1.4	5.0 ± 1.0	4.7 ± 1.0		< 0.001		
HDL-C (mmol/L)	1.6 ± 0.7	1.8 ± 0.5	1.7 ± 0.5		< 0.001		
LDL-C (mmol/L)	3.5 ± 1.9	3.9 ± 1.7	3.7 ± 1.6		0.002		
ΔADP	-10.2 ± 35.3	-8.7 ± 26.6	-12.6 ± 45.1		0.028		
ΔLEP	-0.9 ± 7.1	-0.4 ± 8.1	-1.5 ± 5.1		0.046		
ΔRLTL	-3.1 ± 6.2	-3.1 ± 6.3	-3.0 ± 6.2		0.750		

Abbreviations: ΔRLTL: absolute change of telomere length; BMI: body mass index; WC: waist circumference; HC: hip circumference; SBP: systolic blood pressure; DBP: diastolic blood pressure; TC: total cholesterol; TG: triglycerides; HDL-C: high-density lipoprotein cholesterol; LDL-C: low-density lipoprotein cholesterol; FPG: fasting blood glucose; ΔLEP: absolute change of leptin; ΔADP: absolute change of adiponectin; ΔHOMA-IR: absolute change of Homeostatic Model Assessment for Insulin Resistance; ΔHOMA-β: absolute change of Homeostatic Model Assessment for β-Cell Function; ΔQUICKI: absolute change of Quantitative insulin sensitivity check index; T1: low level; T2: High level

### RLTL levels of the subjects under different grouping conditions

**Fig. 2:**
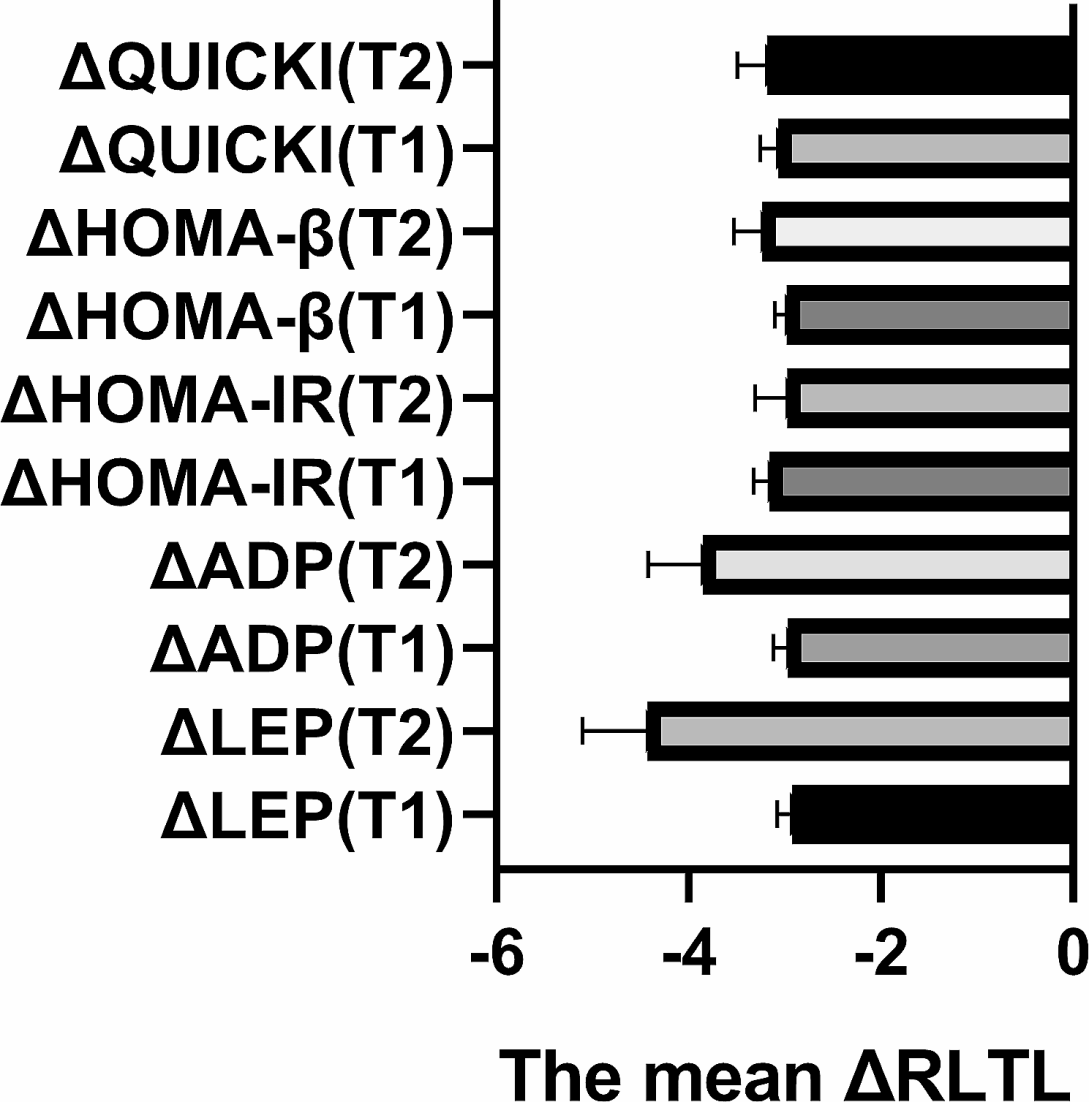
ΔRLTL levels of the subjects under different group. Abbreviations: ΔRLTL, Absolute change of telomere length; ΔLEP: Absolute change of Leptin; ΔADP: Absolute change of Leptin Adiponectin; ΔHOMA-IR: Absolute change of Homeostatic Model Assessment for Insulin Resistance; ΔHOMA-β: Absolute change of Homeostatic Model Assessment for β-Cell Function; ΔQUICKI: Absolute change of Quantitative insulin sensitivity check index; T1: low level; T2: High level

As depicted in Fig. 2, participants were categorized into two groups based on alterations in adipokines, HOMA-IR, HOMA-β, and QUICKI. The analysis revealed that, with the exception of ΔHOMA-IR, the ΔRLTL of the high-level group exhibited a notably lower value compared to that of the low-level group for all other indicators (*P* < 0.05). In other words, RLTL demonstrated a decline with escalating levels of various indicators.

### The relationship between adipose cytokines and RLTL

Table 2 demonstrates the correlation between changes in adipocyte factors and telomere length variations, revealing a negative correlation between ΔLEP and ΔRLTL, with this difference being statistically significant (*P* = 0.004). It is worth noting that even after adjusting for age, the negative correlation between the two remains significant (*P* = 0.009). However, there is no significant correlation between ΔADP and ΔRLTL. Taking into account prior research and other potential factors, we have decided to include ADP in subsequent generalized linear model analyses to further explore the relationship between changes in adipocyte factors and telomere length variations. As shown in Model 1 of Table 3, compared to the low-level group of ΔLEP, the high-level group of ΔLEP exhibits an inverse relationship with ΔRLTL (-1.32 (-2.38, -0.27)), indicating that higher ΔLEP levels correspond to shorter telomere lengths. Even after adjusting for potential confounding factors, the relationship between ΔLEP levels and ΔRLTL persists (-1.31 (-2.24, -0.23), as demonstrated in Model 4 of Table 3). In contrast, there is no relationship between ΔADP and ΔRLTL. When all change values are considered as relative changes rather than delta values, there is no relationship between ΔLEP, ΔADP, and ΔRLTL (see Table S1).

**Table 2:** The analysis of the correlation between changes in telomere length and changes in adipocyte factors

	unadjusted			Age-adjusted	
Variables	Correlation	*P*		Correlation	*P*
Age	0.03	0.400		-	-
ΔADP	-0.05	0.039		-0.05	0.156
ΔLEP	-0.08	0.004		-0.08	0.009

Abbreviations: ΔLEP: absolute change of Leptin; ΔADP: absolute change of leptin;

**Table 3:** The relationship between ΔLEP, ΔADP and ΔRLTL: Results of GLMs with linear regression analysis

Variables	Model 1			Model 2			Model 3			Model 4		
	B (95% CI)	*P*		B (95% CI)	*P*		B (95% CI)	*P*		B (95% CI)	P	
ΔLEP												
T1	1 (Reference)			1 (Reference)			1 (Reference)			1 (Reference)		
T2	-1.32 (-2.38, -0.27)	0.014		-1.31 (-2.37, -0.26)	0.015		-1.32 (-2.38, -0.26)	0.014		-1.31 (-2.24, -0.23)	0.017	
ΔADP												
T1	1 (Reference)			1 (Reference)			1 (Reference)			1 (Reference)		
T2	-0.35 (-1.29, 0.59)	0.464		-0.39 (-1.32, 0.56)	0.431		-0.37 (-1.32, 0.56)	0.430		-0.51 (-2.97, 0.21)	0.264	

Abbreviations: B: partial regression coefficient; β: Standardized regression coefficients; CI: confidence interval; ΔLEP: absolute change of Leptin; ΔADP: absolute change of leptin adiponectin

Model 1: no adjusted; Model 2: Model 1 + sex, age; Model 3: Model 2 + education, smoking, drinking, physical exercise; Model 4: Model 3 + BMI, SBP, DBP, FPG, TG, TC, LDL-C, HDL-C.

### The roles of HOMA-IR, HOMA-β and QUICKI in the relationship between LEP and RLTL

**Fig. 3:**
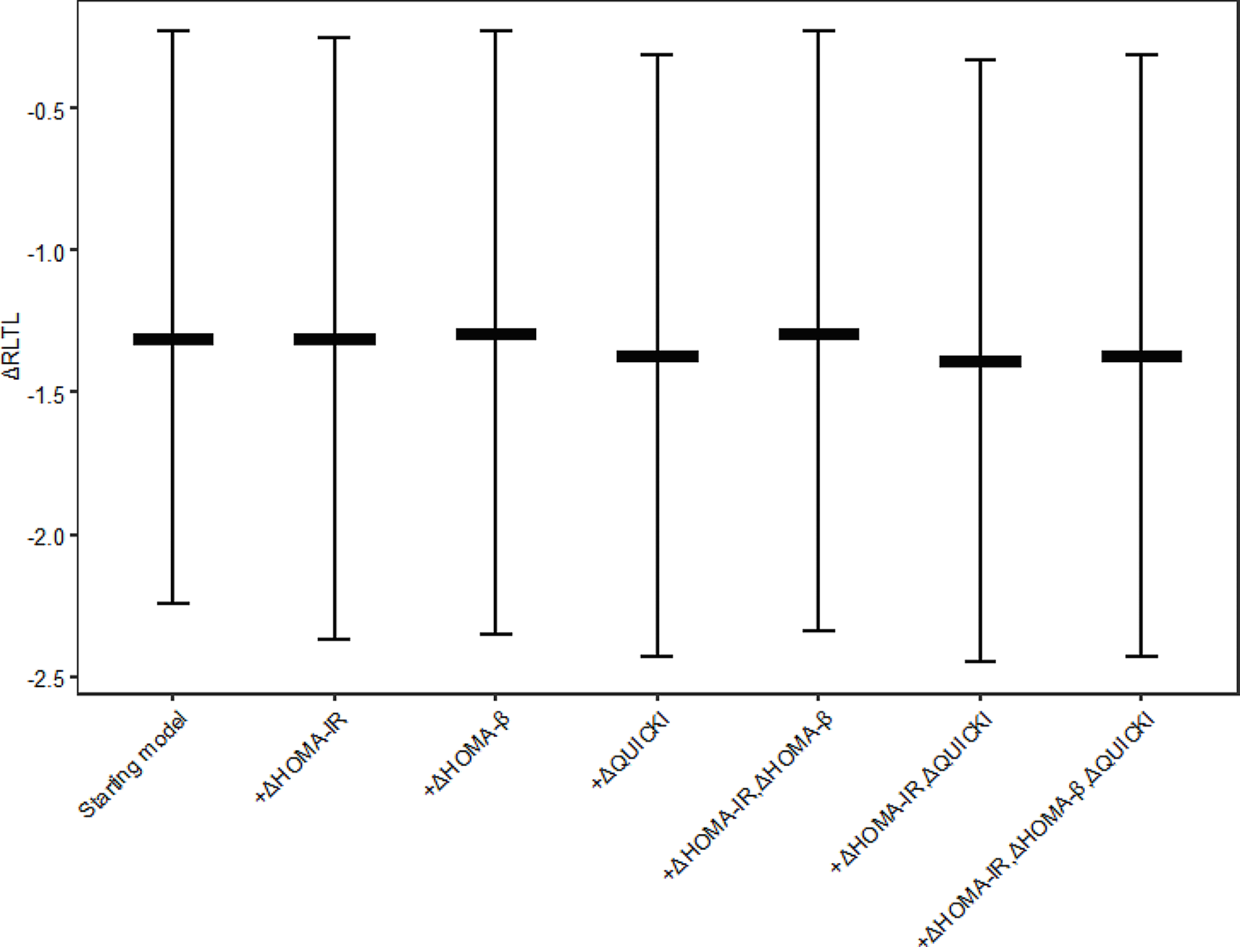
The relationship between ΔLEP and ΔRLTL after adjusting for HOMA-IR, HOMA-β and QUICKI. Abbreviations: B: partial regression coefficient; *CI*: confidence interval, HOMA-IR: Homeostatic Model Assessment for Insulin Resistance; HOMA-β: Homeostatic Model Assessment for β-Cell Function; QUICKI: Quantitative insulin sensitivity check index.; LEP: Leptin; ADP: adiponectin

As shown in Fig. 3, after adjusting for ΔHOMA-IR, high levels of ΔLEP were found to be negatively correlated with telomere shortening compared to the low-level group (B=-1.31, 95% CI (-2.37, -0.25)). Subsequently, after separately adjusting for ΔHOMA-β and ΔQuicki, consistent results were obtained. Furthermore, after simultaneously adjusting for ΔHOMA-IR, ΔHOMA-β, and ΔQuicki, high levels of ΔLEP exhibited a significant negative correlation with ΔRLTL compared to the low-level group (B=-1.37, 95% CI (-2.43, -0.31)). Following this, to further explore whether different steady-state assessment indicators play a role in the relationship between ΔLEP and ΔRLTL, we analyzed the interaction effects of ΔHOMA-IR, ΔHOMA-β, and ΔQuicki on ΔLEP and ΔRLTL. As shown in Table 4, we did not observe significant interaction between ΔHOMA-IR, ΔHOMA-β, ΔQuicki, and ΔLEP (*P* > 0.05). In other words, the interaction between them does not affect ΔRLTL.

**Table 4:** The interaction between ΔLEP and ΔHOMA-IR, ΔHOMA-β, ΔQUICKI influences ΔRLTL (*n* = 1624)

Variables	Model 1			Model 2			Model 3			Model 4		
	F	*P*-value		F	*P*-value		F	*P*-value		F	*P*-value	
ΔLEP	7.916	0.005		5.934	0.015		6.116	0.014		6.394	0.012	
ΔHOMA-IR	0.525	0.469		0.807	0.370		0.857	0.355		0.826	0.364	
ΔHOMA-β	0.998	0.318		0.696	0.405		0.668	0.414		0.352	0.553	
ΔQUICKI	0.214	0.644		0.094	0.759		0.121	0.728		0.054	0.817	
ΔLEP * ΔHOMA-IR	0.257	0.612		0.185	0.668		0.154	0.695		0.144	0.704	
ΔLEP * ΔHOMA-β	1.790	0.182		1.809	0.179		1.841	0.176		1.677	0.196	
ΔLEP * ΔQUICKI	0.001	0.995		0.017	0.896		0.036	0.850		0.053	0.817	

Abbreviations: ΔLEP: absolute change of leptin; ΔHOMA-IR: absolute change of Homeostatic Model Assessment for Insulin Resistance; ΔHOMA-β: absolute change of Homeostatic Model Assessment for β-Cell Function; ΔQUICKI: absolute change of Quantitative insulin sensitivity check index. Model 1: no adjusted; Model 2: Model 1 + sex, age; Model 3: Model 2 + education, smoking, drinking, physical exercise; Model 4: Model 3 + BMI, SBP, DBP, FPG.

## Discussion

In this population-based cohort study, we observed a negative correlation between ΔLEP and ΔRLTL, suggesting that elevated LEP levels over time are associated with shortened telomere length. Moreover, we noted a more pronounced negative correlation between LEP and RLTL after adjusting for ΔHOMA-IR, ΔHOMA-IR, and ΔQUICKI. However, the interaction between ΔLEP and ΔHOMA-IR, ΔHOMA-β, ΔQUICKI did not affect ΔRLTL.

An increasing number of individuals are focusing on factors influencing telomere length and consequently, longevity. It is widely acknowledged that adipose tissue can accelerate aging and contribute to the onset and progression of chronic diseases such as type 2 diabetes, cardiovascular disease, and cancer [17]. These chronic ailments may hasten cell turnover rates, consequently accelerating telomere shortening. Research has highlighted adipose tissue’s capability to secrete various hormones and cytokines, including adiponectin, leptin, IL-6, and TNF-α [18]. Leptin and adiponectin, key adipocyte-derived peptides, exhibit certain associations with telomere length. For instance, Aviv et al. discovered a negative correlation between leptin and leukocyte telomere length in premenopausal women [15]. Another meta-analysis encompassing seven cohorts revealed a negative correlation between leptin and relative telomere length, whereas adiponectin and telomere length showed no significant association [8], consistent with our findings. Nonetheless, some studies contradict our results; for example, one study reported a positive correlation between leptin and telomere length in the elderly [19]. However, many studies have found that leptin and adiponectin are not associated with telomere length [20, 21]. The observation of shorter telomere length in the high-level group of ΔLEP may be attributed to the following reasons. Firstly, leptin plays a crucial role in regulating appetite and energy metabolism, and its elevation may ultimately lead to overweight or obesity. With the increase in body weight, leptin levels may further rise, thereby increasing the risk of low-grade inflammation, which could negatively impact telomere length and result in its shortening [22]. Secondly, dietary patterns vary significantly among regions and populations, and different dietary habits have been linked to telomere length to some extent [23, 24]. Research indicates that leptin, known as the satiety hormone and pro-inflammatory adipocyte factor, is associated with traditional dietary patterns, whereas adiponectin, termed an anti-inflammatory factor, is linked to meat-based dietary patterns and processed foods. Variations in dietary patterns influence changes in telomere length by impacting the levels of leptin and adiponectin [25]. Furthermore, apart from dietary factors, genetic factors also exert a significant influence on telomere length. Leptin, often referred to as the obesity gene, is situated at 7q31.3 and plays a crucial role in regulating obesity by suppressing food intake and enhancing energy expenditure. Mutations in the gene encoding leptin can lead to severe obesity in both animals and humans, with severe obesity potentially resulting in telomere shortening [14]. Lastly, lifestyle factors such as physical activity and stress are also regarded as potential regulatory factors of telomere length. Variations in these factors across different regions and studies may contribute to the onset of various diseases, such as metabolic syndrome, thereby further accelerating telomere shortening [26, 27].

Insulin resistance denotes the condition where normal insulin levels fail to elicit downstream metabolism, resulting in reduced tissue sensitivity to typical insulin concentrations [28]. Even after adjusting for HOMA-IR, we observed an inverse relationship between LEP changes and RLTL. The underlying biological mechanism may be associated with adipocyte secretion implicated in insulin resistance [29]. LEP, a cytokine exacerbating insulin resistance with pro-inflammatory properties, could contribute to accelerated aging. Furthermore, both insulin resistance and leptin are biologically intertwined with inflammation and oxidative stress [30–32]. Studies on this subject have noted that elevated levels of inflammatory and stress markers, such as hsCRP and ANG II, are negatively correlated with TL [7].

However, LEP is not the sole factor influencing telomere length. The aging process in humans is governed by the intricate interplay of various factors, with telomeres serving as a prominent indicator [33]. Consequently, we did not observe any interaction effects between changes in HOMA-IR, HOMA-β, QUICKI, and LEP on telomere length changes. This could be attributed to the fast-paced lifestyle inducing chronic psychological stress in many adults, leading to metabolic disruptions and alterations in the pro-inflammatory biochemical milieu, ultimately culminating in telomere shortening [34]. Telomere length attrition stems from the cumulative impact of inflammation and oxidative stress [15]. Additionally, factors like racial disparities, study design, and sample size may potentially influence the relationship between telomere length and insulin resistance. Furthermore, given the examination of changes in insulin resistance’s effects on telomeres across various timeframes in this study, diverse outcomes are likely. Alongside variations in the study population and inclusion/exclusion criteria, further investigation is warranted to elucidate the mechanism underlying the positive association of RLTL with insulin resistance.

### Strengths and limitations

The present study boasts several notable strengths. Firstly, we employed a longitudinal study design to scrutinize changes over time. Moreover, by computing alterations in insulin resistance and inflammatory markers, we achieved a more comprehensive characterization of temporal variations. Additionally, interaction analysis was conducted to explore the mediating role of adipokines in the relationship between changes in insulin resistance and relative telomere length. However, our study has several limitations. Firstly, while the gold standard for assessing insulin resistance is the glucose clamp technique, we utilized HOMA-IR due to its feasibility in large study populations [35]. Although HOMA-IR, derived from blood glucose and insulin levels, has served as a reliable indicator for evaluating insulin resistance in numerous population-based studies, it may not capture the full complexity of the phenomenon [36]. Secondly, the limited sample size of our study necessitated the measurement of RLTL instead of LTL, potentially leading to differences in the results. Thirdly, the significant correlation observed between changes in insulin resistance and adipocytokines may vary among study participants, warranting further research to thoroughly investigate these effects over an extended period and ascertain their generalizability to other cohorts. Fourthly, telomere length is influenced by age, and discrepancies among different cohorts could stem from variations in age distribution. Additionally, residual confounding factors, such as medication use, may exist, underscoring the need for meticulous consideration in future research endeavors. Moreover, misclassification errors, particularly with categorical variables, may introduce discrepancies with other findings. Furthermore, the long-term duration of the study may subject it to the effects of aging, potentially impacting the primary research outcomes. Lastly, telomere length is likely influenced by diet and genetic factors. However, due to baseline limitations, detailed information regarding these variables could not be obtained, precluding definitive conclusions about their specific impact on telomere length.

## Conclusion

This study observed a significant correlation between the increase in Leptin levels and the gradual shortening of telomere length. This correlation was further strengthened after incorporating HOMA-IR, HOMA-β, and QUICKI into the model. However, the interaction effects between HOMA-IR, HOMA-β, QUICKI, and LEP respectively had no impact on RLTL. These findings suggest that Leptin may influence overall health status through its effect on telomere length, thereby necessitating the development of measures aimed at reducing Leptin levels to prevent the onset and progression of related diseases.

### Electronic supplementary material

Below is the link to the electronic supplementary material.

Supplementary Material 1

## Data Availability

No datasets were generated or analysed during the current study.

## Abbreviations

BMI: Body mass index
FPG: Fasting blood glucose
HDL-C: High density lipoprotein cholesterol
LDL-C: Low density lipoprotein cholesterol
TC: Total cholesterol
TG: Triglyceride
SBP: systolic blood pressure
DBP: diastolic blood pressure
WC: Waist circumference
LTL: leukocyte telomere length
HOMA-IR: Homeostatic Model Assessment for Insulin Resistance
FINS: Serum insulin concentrations
RLTL: Relative leukocyte telomere length
ADP: adiponectin
HOMA-β: Homeostatic Model Assessment for β-Cell Function
QUICKI: Quantitative insulin sensitivity check index
*CI*: confidence interval
