# Supplementary material for: Study of association of leptin with leukocyte telomere length in a Chinese rural population
Source: Lipids Health Dis. 2024 Apr 13;23:103. doi: 10.1186/s12944-024-02097-x (PMC11016218; doi:10.1186/s12944-024-02097-x)
Supplement: Supplementary file 1 — Supplementary Material 1 [file 12944_2024_2097_MOESM1_ESM.docx]

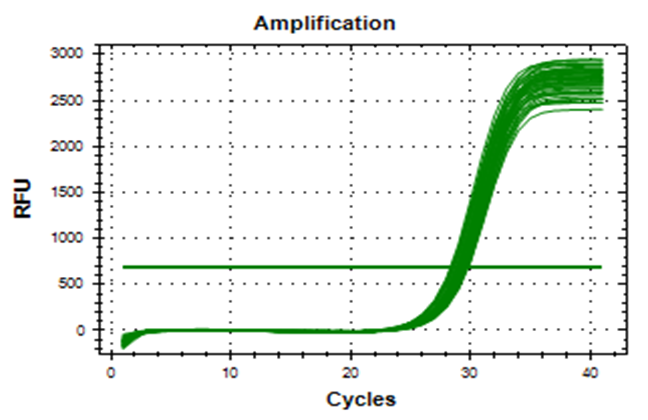


Figure S1 The RT-PCR amplification curve of relative telomere length in peripheral blood leukocytes.


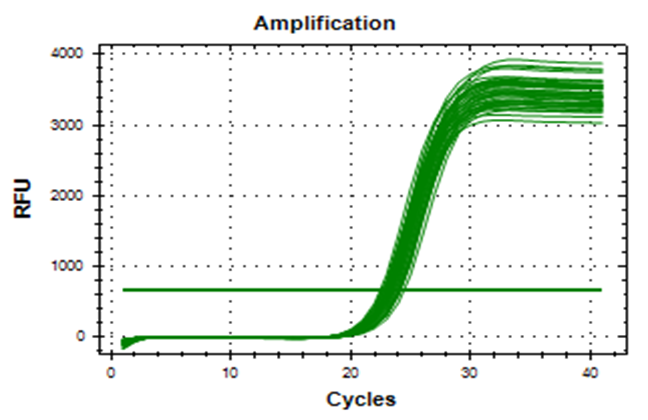


Figure S2 The RT-PCR amplification curve graph of the reference gene.

Table S1 The relationship between ΔLEP, ΔADP and ΔRLTL: the change value is relative change.

| Variables | Model 1 | |  | Model 2 | |  | Model 3 | |  | Model 4 | |  |
| --- | --- | --- | --- | --- | --- | --- | --- | --- | --- | --- | --- | --- |
|  | B (95% *CI*) | *P* |  | B (95% *CI*) | *P* |  | B (95% *CI*) | *P* |  | B (95% *CI*) | *P* |  |
| ΔLEP |  |  |  |  |  |  |  |  |  |  |  |  |
| T1 | 1 (Reference) |  |  | 1 (Reference) |  |  | 1 (Reference) |  |  | 1 (Reference) |  |  |
| T2 | -6.138 (-13.512, 1.236) | 0.103 |  | -4.866 (-12.590, 2.858) | 0.217 |  | -5.034 (-12.831, 2.762) | 0.206 |  | -4.984 (-12.787, 2.890) | 0.216 |  |
| ΔADP |  |  |  |  |  |  |  |  |  |  |  |  |
| T1 | 1 (Reference) |  |  | 1 (Reference) |  |  | 1 (Reference) |  |  | 1 (Reference) |  |  |
| T2 | -1.895 (-9.269, 5.479) | 0.614 |  | -1.502 (-8.927, 5.922) | 0.692 |  | -1.042 (-8.496, 6.411) | 0.784 |  | -1.288 (-8.807, 6.231) | 0.737 |  |

Abbreviations: B: partial regression coefficient; β: Standardized regression coefficients; *CI*: confidence interval, ΔLEP: relative change of leptin; ΔADP: relative change of adiponectin.

Model 1: no adjusted; Model 2: Model 1+sex, age; Model 3: Model 2+education, smoking, drinking, physical exercise; Model 4: Model 3 + BMI, SBP, DBP, FPG, TG, TC, LDL-C, HDL-C.
